# Towards an Integrated Approach for Monitoring Toxoplasmosis in Southern Italy

**DOI:** 10.3390/ani11071949

**Published:** 2021-06-30

**Authors:** Paola Pepe, Antonio Bosco, Federico Capuano, Loredana Baldi, Angela Giordano, Andrea Mancusi, Marialuisa Buonanno, Luigi Morena, Renato Pinto, Paolo Sarnelli, Giuseppe Cringoli, Laura Rinaldi

**Affiliations:** 1 Department of Veterinary Medicine and Animal Production, University of Naples Federico II, 80137 Naples, Italy; boscoant@tiscali.it (A.B.); cringoli@unina.it (G.C.); lrinaldi@unina.it (L.R.); 2 Centro Regionale per il Monitoraggio delle Parassitosi (CReMoPAR), Regione Campania, 84025 Eboli, Salerno, Italy; 3 Istituto Zooprofilattico Sperimentale del Mezzogiorno, 80055 Portici, Naples, Italy; federico.capuano@cert.izsmportici.it (F.C.); l.baldi@izsmportici.it (L.B.); angela.giordano@cert.izsmportici.it (A.G.); andrea.mancusi@izsmportici.it (A.M.); marialuisa.buonanno@izsmportici.it (M.B.); 4 Centro di Riferimento Regionale Sanità Animale (CReSan), Regione Campania, 84127 Salerno, Italy; l.morena@aslsalerno.it; 5 UOD Prevenzione e Sanità Pubblica Veterinaria Regione Campania, 80143 Naples, Italy; renato.pinto@regione.campania.it (R.P.); p.sarnelli@regione.campania.it (P.S.)

**Keywords:** *Toxoplasma gondii*, livestock, monitoring, risk factors, humans, Italy, One Health

## Abstract

**Simple Summary:**

Toxoplasmosis is a significant public health issue worldwide, caused by the intracellular protozoan *Toxoplasma gondii*. It has a heteroxenous life cycle in which felines act as definitive reservoirs and a wide range of warm-blooded animals, including humans, act as intermediate hosts. Due to the complex life cycle, monitoring, prevention and control of this parasite are very difficult. A thorough analysis of the epidemiology of *T. gondii* in humans, animals and food as well as the risk factors associated with the infection are needed to plan adequate control strategies in a given geographical area. Based on this, an integrated approach for monitoring toxoplasmosis was developed and conducted in an endemic area of southern Italy. The main tasks of this approach were based on the following strategies: parasitological and risk factor analysis for *T. gondii* in livestock farms, serological and molecular monitoring in meat-producing livestock at slaughterhouses, hospital discharge records (HDRs) analysis and outreach activities. The findings of this study confirmed the spread of *T. gondii* infection in southern Italy with high prevalence values in ruminants and the need of valid control strategies based on comprehensive and transdisciplinary actions according to the One Health approach.

**Abstract:**

Toxoplasmosis is a widespread worldwide zoonotic infection caused by the intracellular protozoan *Toxoplasma gondii*. This protozoan infection is considered one of the most important food-borne parasitic zoonoses globally. Beyond its impact on public health, toxoplasmosis has also important veterinary implications, because it causes miscarriage or congenital malformations in livestock with negative economic impacts. An integrated monitoring programme aimed to deepen the epidemiological data on toxoplasmosis and to identify the risk factors that may favour *T. gondii* infections in animals and humans was conducted in an endemic area of southern Italy. The monitoring activities were based on the following tasks: (i) parasitological analysis and risk factors for *T. gondii* in livestock (sheep, goat, cattle and water buffalo) farms; (ii) serological and molecular monitoring at slaughterhouse in meat-producing livestock; (iii) analysis of hospital discharge records (HDRs); (iv) outreach activities (information, dissemination and health education) to farmers, vet practitioners and school-age children. The present study confirmed a very high seroprevalence of *T. gondii* infection in livestock farms (e.g., up to 93.1% in sheep farms) in southern Italy and highlighted the potentially significant public health risk in this area.

## 1. Introduction

Toxoplasmosis is a zoonotic infection with a worldwide distribution caused by the intracellular protozoan *Toxoplasma gondii*. The definitive hosts of *T. gondii* are felids, and these play an essential role in the contamination of the environment with oocysts, whereas a wide range of warm-blooded animals, including humans, act as intermediate hosts [1]. Toxoplasmosis is considered one of the most important food-borne parasitic zoonoses globally [2]. Based on the disease burden, the WHO Foodborne Disease Burden Epidemiology Reference Group (FERG) listed the prioritised food-borne parasites, of these *T. gondii* resulted third with 1.7 million Disability Adjusted Life Years—DALYs [3]. Most infections appear to be asymptomatic in immunocompetent persons; however, the parasite can cause serious disease in humans, especially neonates [4], and immunocompromised people, who are at a risk of developing cerebral toxoplasmosis [5]. Among the several ways of transmission, the consumption of food/water contaminated with oocysts dispersed by cats and other felines, raw/undercooked meat containing tissue cysts or un-pasteurized milk containing tachyzoites, as well as the transplacental route are the most common [6,7].

Beyond its impact on public health, *T. gondii* infection also has important veterinary implications especially in small ruminants where the protozoa mainly affect the reproductive organs resulting in abortions, fetal mummifications, stillbirths and the birth of weak offspring. Hence, toxoplasmosis may have severe negative socio-economic effects on veterinary and public health [8]. 

Due to the complex life cycle, monitoring, prevention and control of this parasite are very difficult and require a comprehensive and transdisciplinary approach [9]. A thorough analysis of the epidemiology of *T. gondii* in humans, animals and food as well as the assessment of risk factors associated with the infection are needed in order to plan adequate control strategies in a given geographical area [10]. For this purpose, an accurate diagnosis, using highly sensitive and specific methods, is crucial. So far, diagnostic tools available to detect *T. gondii* infection in livestock have included direct (e.g., histopathology, immunohistochemistry, polymerase chain reaction—PCR and bioassays) and indirect methods (serological tests based on the detection of antibodies against the parasite) [11]. Currently, prevalence studies in livestock are mainly based on serological analysis, of these the enzyme-linked immunosorbent assay (ELISA) test is the most cost-effective and convenient diagnostic tool for large-scale surveys although the immunofluorescence antibody test (IFAT) is considered the gold standard. Recently, meat juice has been also suggested as serological matrix for improving meat inspection because to date, the PCR methods still show a low sensitivity [12].

Several studies have been conducted to establish the seroprevalence of *T. gondii* in livestock across the Italian regions. In central-northern Italy, seroprevalence ranged from 27.5% to 60.6% at individual animal level [13,14] and up to 97.0% [13] at farm level in small ruminants whereas a prevalence value of 68.4% was reported in cattle farms [15]. The same epidemiological scenario was encountered in southern and insular regions, where high prevalence values, up to 87.0%, were reported in small ruminant farms [16]. In the Campania region of southern Italy, prevalence values in livestock have been reported as follows: 77.8% in sheep [17] and 13.7% in water buffalo farms [18], respectively. Furthermore, high prevalence values (39.6%) were recently found in wild boars [19]. 

So as to reduce the regional spread of toxoplasmosis, since 2019, the Campania government supported and financed “ToxoCamp”, a monitoring programme that, through a multi-institutional approach, aimed at deepening the epidemiological data on toxoplasmosis and to identify the risk factors which may favour *T. gondii* infections in animals and humans. 

This paper describes the main activities and findings of the ToxoCamp programme, highlighting the strategies of actions used in this endemic region of southern Italy. The final goal was to evaluate the real impact of this zoonosis to develop adequate control strategies according to the One Health concept. 

## 2. Materials and Methods

### 2.1. Study Design

The activities of ToxoCamp were performed from January 2019 to December 2020 in the Campania region. The main tasks carried out in this programme were: (i) parasitological analysis and risk factors for *T. gondii* in ruminant livestock (sheep, goat, cattle and water buffalo) farms; (ii) serological and molecular monitoring at slaughterhouses in meat-producing livestock; (iii) analysis of hospital discharge records (HDRs); (iv) outreach activities (information, dissemination and health education) to farmers, vet practitioners and school-aged children.

### 2.2. Task 1. Parasitological Analysis and Risk Factors for T. gondii in Ruminant Livestock Farms

#### 2.2.1. Selection of Livestock Farms

A total of 104 livestock farms (29 sheep, 26 goat, 25 cattle and 24 water buffalo farms) were selected using simple random sampling to ensure a representative sample of farms from the Campania region. Sample size was calculated considering the following parameters: population size, expected farm-prevalence (70%), absolute error (8%) and confidence level (95%) [20]. The calculations determined that a minimum of 96 farms should be sampled for this study.

#### 2.2.2. Serological Analysis (Livestock)

In each farm, blood samples were collected from 15 adult (older than 18 months) and 5 young (4–18 months) animals (when possible). Blood samples were transferred to the laboratory on ice. After centrifugation (1690× *g* for 10 min), the sera were stored at −20 °C until analysis. Sera samples were tested for *T. gondii* antibodies by a commercial ELISA kit (ID Screen^®^ Toxoplasmosis Indirect Multi-Species, IDVET, Montpellier, France) according to the manufacturer’s instruction. Positive and negative sera provided with the kit were used as controls. For each sample, the resulting values were calculated by applying the formula supplied in the kit: S/P% = OD sample − OD-negative control/OD-positive control − OD-negative control) × 100. Samples with S/P% ≥ 50% were considered positive.

#### 2.2.3. Serological and Copromicroscopic Analysis (Cats) 

**Figure 1:**
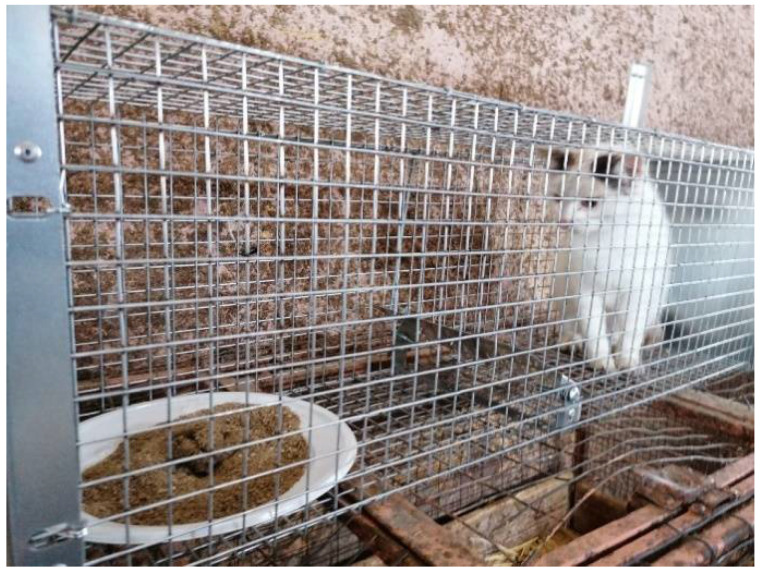
Confinement of a cat by using a trap cage.

In each farm whose livestock resulted serologically positive to *T. gondii* (see the results section), the cats present were subjected to parasitological examination. Trap cages for cats (https://www.cage-system.com/trappole/ accessed date: 15 June 2021) were used to allow the confinement of cats for 48 h respecting the standards on animal welfare and safety of staff (Figure 1). 

After 48 h, blood and faecal samples were collected, and cats were released. The blood samples were processed and analysed using the commercial ELISA kit (ID Screen^®^ Toxoplasmosis Indirect Multi-Species, IDVET, Montpellier, France) as described above. Copromicroscopic exams were performed using the FLOTAC dual technique [21], with sodium chloride (specific gravity – s.g. = 1.20) and zinc sulphate (s.g. = 1.20) as flotation solutions with a detection limit of 2 oocysts per gram of faeces.

#### 2.2.4. Questionnaire Data Collection

A questionnaire was prepared to include questions on different management variables (type of production, number of animals, presence of other domestic animals at farms, presence of resident and/or stray cats, frequency of grazing, transhumance and presence of any control rodent measures) related to farm and pasture typology. The questionnaire was administered by the farm veterinarian at the time of blood sample collection to all participating farmers. 

#### 2.2.5. Statistical Analysis

The differences in seroprevalence among the different animal species were analysed by the Chi-square test. Multivariate logistic regression models were used to identify the risk factors for *T. gondii* seropositivity in livestock farms. Each model was applied at farm level, using all the data recorded (e.g., management variables related to farm and pasture typology; presence of cats and control rodent measures) as independent variables and the *T. gondii* serological status (positive/negative), as dependent variable. The odds ratio (OR) was used to estimate the strength of the association between each factor included in the study and the positive status to *T. gondii*. The independent variables considered in the final model were those showing probabilities <0.05. All the statistical data were analyzed using a dedicated software (SPSS, Version 22.0, IBM Corporation, Armonk, NY, USA).

### 2.3. Task 2. Serological and Molecular Monitoring in Meat-Producing Livestock at slaughterouses

Overall, 193 adult animals (50 sheep, 50 goats, 45 cattle and 48 water buffaloes), slaughtered in five sentinel abattoirs located in the Campania region, were randomly sampled. In addition, a total of 218 pigs, during at-home animal slaughter, were investigated.

For each animal slaughtered, a blood sample was collected from the jugular vein into tubes without anticoagulants, and approximately 50 g of myocardium and diaphragm tissue samples, respectively, were collected and placed into plastic bags suitable for biological samples. Blood and tissue samples were transported to the laboratory within a few hours; blood was centrifuged (1690× *g* for 10 min), and serum was transferred into Eppendorf tubes and stored at −20 °C until further serological analysis. Each tissue sample, either myocardium or diaphragm, was divided into two aliquots. The first aliquot (25 g) was frozen in a plastic bag overnight, subsequently thawed to extract meat juice, and stored at −20 °C until serological analysis. The second aliquot (25 g) was stored at −20 °C until molecular analysis. 

#### 2.3.1. Serological Analysis

Serum and meat juice samples were analyzed for *T. gondii* antibodies with a commercial ELISA kit (ID Screen^®^ Toxoplasmosis Indirect Multi-Species, IDVET, Montpellier, France), according to the manufacturer’s instruction (see above), using two different dilution ratios, i.e., 1:10 and 1:2, for serum and meat juice samples, respectively.

#### 2.3.2. K-Agreement

The Cohen’s ĸ value, for each animal species, was calculated to evaluate the agreement among different biological matrices (serum, meat juices from myocardium and diaphragm, respectively) used in the ELISA test. The κ values obtained were interpreted as follows: slight agreement (ĸ < 0.2); fair agreement (ĸ = 0.2–0.4); moderate agreement (ĸ = 0.4–0.6); good agreement (ĸ = 0.6–0.8); or very good agreement (ĸ > 0.8) [20]. All statistical analyses were performed using the SPSS software (version 22.0, IBM Corporation, Armonk, NY, USA) and the significance level was set at a *p*-value of ≤0.05.

#### 2.3.3. Molecular Analysis

Twenty-five grams of each sample were homogenized by means of a stomacher (Interscience, France) and then 25 mg of each lysate sample were subjected to DNA extraction with the commercial kit QIAamp DNA Mini (Qiagen, Hilden, Germany).

The real-time PCR was used to detect the 529 bp RE gene target of *T. gondii* in a final reaction volume of 20 µL. Briefly, 2 μL of template DNA was added to a reaction mixture containing: 10 μL of TaqMan universal master mix, 500 nM of each primer (forward primer AF1, 5′-CACAGAAGGGACAGAAGT-3′ and reverse primer AF2, 5′-TCGCCTTCATCTACAGTC-3′), 250 nM of TaqMan probe (FAM-5CTCTCCTCCAAGACGGCTGG-BHQ-3′), 2 μL of Exo IPC Mix, 0.4 μL of Exo IPC DNA and H_2_O to a final volume of 20 μL. The thermal profile was as follows: 50 °C for 2 min, 95 °C for 10 min, 40 cycles: 95 °C for 15 s and 60 °C for 1 min PCR amplifications were performed on CFX96 DeepWell (Bio-Rad, Hercules, CA, USA) [22].

### 2.4. Task 3. Hospital Discharge Records (HDRs) Analysis

The hospital discharge records (HDRs) data from 2009 to 2013, in anonymous form and free of personal information, were provided by the Italian Department of Health. The HDRs contained an anonymous individual code for tracking patient’s hospital admissions, discharges, and readmissions. Each anonymous individual code identified one patient. Specifically, the database included: patient’s admission and discharge dates, gender, age, domicile code, type of hospitalization (ordinary hospitalization or day hospital).

### 2.5. Task 4. Outreach Activities

Dissemination meetings as well as webinars for farmers, veterinary practitioners and school-age children were organized throughout the duration of the programme. The objectives of these activities were focused mainly on providing key information regarding: (i) the parasite life cycle; (ii) actions to avoid infection in the animal and human population. In addition, educational materials (brochures and posters) were designed to support the activities of dissemination (Supplementary Materials). 

## 3. Results

### 3.1. Task 1. Parasitological Analysis and Risk Factors for T. gondii in Ruminant Livestock Farms

**Figure 2:**
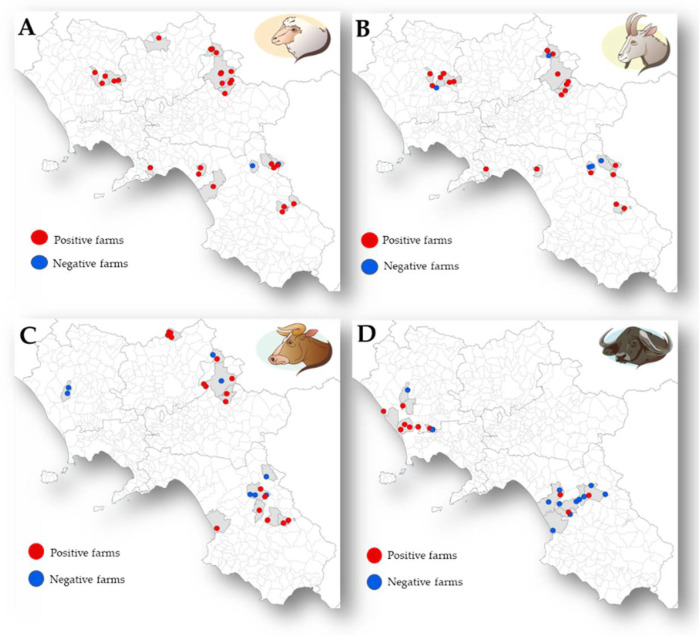
Distribution of *T. gondii* in the livestock farms analysed according to animal species: (**A**) sheep; (**B**) goats; (**C**) cattle and (**D**) water buffaloes.

Serum samples were collected from 1127 animals in 104 farms. A total of 426 animals (37.8%; 95% Confidence Interval [CI] = 35.0–40.7) resulted positive for *T. gondii* with the highest mean S/P% values in sheep and goats (142% and 163%, respectively), while the mean S/P% values in cattle and water buffaloes were 93% and 59%, respectively. Seroprevalence of *T. gondii* varied significantly (*p* < 0.05) among ruminant species showing higher values in small (sheep and goats) than in large (cattle and water buffaloes) ruminants. The distribution of *T. gondii* in the livestock farms analysed is showed in Figure 2. The prevalence obtained from the serological analysis, both at farm and animal level, are showed in Table 1. Furthermore, Table 2 reports the seroprevalence according to the age of animals (youngs and adults) for each ruminant species analysed.

In the 76 farms whose ruminants resulted serologically positive to *T. gondii*, a total of 304 cats were investigated. Of these, 298 cats (98.0%; 95%CI = 95.5–99.2) resulted positive for *T. gondii* antibodies whereas all of them were negative for *T. gondii* oocysts (0.0%).

#### Statistical Analysis

Depending on the animal species, the multivariate logistic regression analysis identified a strong association between the seropositivity to *T. gondii* and the variables “presence of cats” and “abortion” (*p* < 0.05). In contrast, the variables “presence of young animals” and “rodent control measures” were associated with a significant low *T. gondii* seroprevalence (Table 3). 

### 3.2. Task 2. Serological and Molecular Monitoring of Meat-Producing Livestock at Slaughterouses 

Blood, myocardium and diaphragm tissue samples were collected from a total of 411 animals. The prevalence values obtained from serological analysis for each animal species were: 96.0%, 98.0%, 17.8%, 8.3% and 5.5% in sheep, goats, cattle, water buffaloes and pigs, respectively.

Table 4 shows the results obtained from serological and molecular analysis, for each matrix and according to animal species.

#### K-Agreement

The agreements, resulted from the *T. gondii* antibody detection using different serological matrices (i.e., serum, meat juice from myocardium, meat juice from diaphragm), are shown in Table 5.

The best results, with an agreement from good (0.6 < ĸ < 0.8) to very good (ĸ ≥ 0.8), were obtained between serum and meat juice from myocardium for most of the animal species analysed (sheep, goats, water buffaloes and pigs). Only in cattle, a better agreement value was found between serum and diaphragm (ĸ = 0.910) compared to serum and myocardium (ĸ = 0.831).

### 3.3. Task 3. Hospital Discharge Records (HDRs) Analysis

The HDRs analysis showed an incidence of human toxoplasmosis hospitalizations in the Campania region of approximately 0.72/100,000 inhabitants. Most patients with toxoplasmosis aged less than 1 year, followed by adults aged between 25 and 44 years. No difference was found regarding their gender.

### 3.4. Task 4. Outreach Activities

A total of 21,000 dissemination materials including brochures and posters about the toxoplasmosis with cartoon pictures tailored for both children and adults were produced and distributed to farmers, veterinarians and school-aged children. 

A total of 20 meetings were organized with primary and middle school students (No. = 5), farmers (No. = 10) and veterinarians (No. = 5) to explain the different aspects of toxoplasmosis, as the disease, the life cycle and the best preventive measures. 

## 4. Discussion

This study shed light on the current epidemiological situation of *T. gondii* in the Campania region of southern Italy, confirming the occurrence of a very high seroprevalence of this infection in livestock farms in southern Italy and highlighting the significant public health risk in this area. 

Toxoplasmosis is widespread and its seroprevalence in livestock vary widely, reaching high values in sheep (98.9%), goats (95.2%), cattle (93.5%) and pigs (96.6%) reviewed in [1,23,24,25]. The seroprevalence rates, at individual and farm level, in livestock reported here are in line with the results from different studies conducted in other Italian regions [13,15,16,26]. According to these studies, a higher seroprevalence was recorded in small ruminants (sheep and goats) than in cattle and water buffaloes due probably to differences in susceptibility to *T. gondii* infection and to differences in farm management.

Since in ruminants the principal route of infection with *T. gondii* appears to be the ingestion of oocysts contaminating feed, water or the environment, control strategies for stray cat populations must be implemented [27]. To date, the seroprevalence values of *T. gondii* in domestic cats worldwide range between 10.0% and 84.7% [28]. In Italy, the seroprevalence observed in privately owned and stray cats was 42.3% [29] and 30.5% [30], respectively. Comparing with these studies, the seroprevalence observed in our survey was higher (98.0%). The different methodologies used, different sample sizes and sample populations surveyed may have contributed to these differences; therefore, it is difficult to compare the reported prevalence. Despite the high seroprevalence, no faecal samples of cats resulted positive to *T. gondii* oocysts, confirming that the copromicroscopic method is not a robust assay for the identification of potentially shedding cats in cross-sectional surveys [29]. Indeed, cats have been shown to excrete *T. gondii* oocysts for a limited and relatively short period when a primary infection takes place. Then, cats usually develop antibodies to *T. gondii* 1–2 weeks after they have shed oocysts [31]. This may be the reason why oocysts were not detected in the faecal samples of all the seropositive cats examined in the present study.

In order to reduce the risk of human infection with *T. gondii*, the knowledge of potential risk factors associated with the infection of farm animals with the parasite is of fundamental importance to implement the Hazard Analysis and Critical Control Points (HACCP), allowing the farmers to develop efficient and sustainable control measures against *T. gondii* infection in their farms [2].

In this study, the multivariate logistic regression model identified the presence of outdoor domestic cats as a risk factor for infection with *T. gondii* in farm animals, especially in sheep and goat farms, as previous reported by other authors [11,13,32]. Interestingly, a strong association between seroprevalence and access of cats to water and not to the feed store was found by Cenci-Goga et al. [13], thus supporting the importance of waterborne transmission of *T. gondii* in sheep farms. In contrast to Cenci-Goga et al. [13], in our study only the presence of stray cats was assessed as significant variable, not their access to water or feed store. Therefore, further analysis should be performed to evaluate the realistic chance of cats to contaminate farmland, feed or water provided to livestock [30].

Young animals in sheep farms were associated with a low seropositivity to *T. gondii*. Indeed, age was widely identified as a risk factor associated with the spread of *T. gondii* infection in numerous studies and it was observed that the seroprevalence increased with the age of the animal most likely due to the time of exposure to the infective stages of the parasite [32], thus suggesting that most infections would occur postnatally [33]. On the other hand, a strong association was found between the variable abortion and the seropositivity to *T. gondii* in goat and in water buffalo farms. The abortive role of *Toxoplasma* in small ruminants is widely recognized [23,24] whereas it is not common in cattle [34] and water buffaloes [35]. Recently, Ciuca et al. [18] showed that the co-infection by *Neospora caninum* and *T. gondii* is significantly associated with abortion in a water buffalo farm located in the same area.

Furthermore, a significant association between the presence of control rodent measures and a low seropositivity to *T. gondii* was found in cattle and water buffalo farms. The association between seropositivity and lacking adequate biosecurity and pest management practices was widely associated to seropositivity to *T. gondii* infection in livestock farms [36,37]. No association to *T. gondii* infection was found with the other farm management factors analysed in this study. Thus, according to the risk factor analysis, particular attention should be given to the hygienic measures and procedures in farms by, for example, keeping indoor animals, denying access to cats at sites of food storage, controlling rodents and other animal pests, and providing clean drinking water to animals. In addition, potential on-farm interventions to control *T. gondii* should include the vaccination of sheep [38].

To gain more insight into the role of meat as a source of human infection with *T. gondii*, in this study, seroprevalence in the main meat-producing livestock species was investigated. According to the results obtained in other studies [14,39,40,41], high seroprevalence values were reported during the slaughter’s activities, highlighting the fundamental role that infected meat plays in the *T. gondii* epidemiology. Of these, the seroprevalence obtained in pigs raised for familiar consumption are of particular importance since the pork products are, usually, consumed raw, processed only by smoking and/or salting and thus, may be potential sources of toxoplasmosis for humans [42]. Furthermore, given the important role of the sylvatic cycle in the spread of the toxoplasmosis, more attention should be paid also to the control of the sylvatic animals such as wild boars, according to the European legislation that included *T. gondii* in the list of zoonotic agents to be subjected to epidemiological monitoring in wildlife [43].

The results of the K-agreement analysis showed that meat juice from myocardium consistently provided the best agreement with serum results in line with previous findings [44,45], hence providing further evidence that meat juice samples can be used in seroprevalence studies where serum or plasma samples cannot be collected [12].

Compared to the total number of seropositive samples, only a few samples were found to be positive using the real-time PCR. This lower sensitivity has been also reported in other studies [12,40,41]. The low detection may be due to the limited amount of sample that can be tested or to the irregular distribution of tissue cysts in muscles and organs. To improve the sensitivity of molecular detection, a magnetic capture-based DNA extraction has been developed and used for testing large amounts of tissue (up to 100 g), increasing the probability of including a portion of tissue containing parasite DNA [46]. In addition, novel technologies, such as the droplet digital polymerase chain reaction (ddPCR) could be used to provide higher sensitivity compared to the real-time PCR [47].

The results obtained from the HDRs analysis evidenced as toxoplasmosis continues to be a public health problem in this area with an incidence of 0.72/100,000 inhabitants. It is important to note that the HDRs did not include cases managed in an outpatient setting or asymptomatic cases, so the data represent only the tip of the iceberg of the real burden of toxoplasmosis. To obtain a more detailed information about delivery and neonatal care in the Campania region, it would be appropriate to collect data from all deliveries included in the Italian Birth Register-CeDAP and integrate them with HDRs.

During the two years of ToxoCamp, outreach activities (information, dissemination and health education) to farmers, vet practitioners and school-age children have been performed to increase the *Toxoplasma*-related knowledge, control and prevention as well as the main risk factors. Indeed, it is well known that the effectiveness of a control program is strongly associated with a good education program addressed to the community and aimed to reduce the risk of toxoplasmosis.

## 5. Conclusions

Reducing *T. gondii* infection in animals is critical to prevent foodborne transmission of *T. gondii* to humans according to the One Health perspective. To increase the feasibility of preventing infection in food animals, screening to identify farms with infected animals should be routinely performed.

Finally, due to the impact of toxoplasmosis on public and veterinary health, a greater institutional awareness of the pathways of infection and comprehensive and transdisciplinary actions to control transmission are needed.

## Figures and Tables

**Table 1:** Overall seroprevalence of *T. gondii* at farm and animal level according to animal species (sheep, goats, cattle and water buffaloes).

Animal Species	No. Farms Analysed	No. Farms Positive	Prevalence (%) (95%CI)	No. Animals Analysed	No. Animals Positive	Prevalence (%) (95%CI)
Sheep	29	27	93.1 (75.8–98.8)	390	221	56.7 (51.6–61.2)
Goats	26	21	80.8 (60.0–92.7)	241	114	47.3 (40.9–53.8)
Cattle	25	17	68.0 (46.4–84.3)	296	48	16.2 (12.3–21.0)
Water buffaloes	24	11	45.8 (26.2–66.8)	200	43	21.5 (16.1–28.0)
TOTAL	104	76	73.1 (63.3–81.1)	1.127	426	37.8 (35.0–40.7)

**Table 2:** Overall seroprevalence of *T. gondii* according to the age (youngs and adults) for each animal species (sheep, goats, cattle and water buffaloes).

Animal Species	Age	No. Animals Analysed	No. Animals Positive	Prevalence (%) (95%CI)
Sheep	Youngs Adults	78 312	32 190	41.0 (30.2–52.7) 60.9 (55.2–66.3)
Goats	Youngs Adults	39 202	17 97	43.6 (28.2–60.2) 48.0 (41.0–55.1)
Cattle	Youngs Adults	62 234	19 42	30.6 (19.9–43.8) 17.9 (13.4–23.6)
Water buffaloes	Youngs Adults	9 191	0 43	0.0 22.5 (16.9–29.2)

**Table 3:** Results of the multivariate logistic regression analysis. Variables associated with seropositivity to *T. gondii*, according to the animal species.

Animal Species	Variable	Standard Error	Wald’s Chi-Square	Odds Ratio	*p*-Value
Sheep	Young sheep (<12 months)	0.592	5.852	0.239	0.016
	Presence of cats	0.341	11.381	3.164	0.001
Goats	Presence of cats	0.308	15.074	3.306	0.000
	Abortion	0.284	6.502	2.064	0.011
Cattle	Control rodent measures	0.608	13.602	0.106	0.000
Water buffaloes	Abortion	0.476	4.796	2.837	0.029
	Control rodent measures	0.792	35.350	0.009	0.000

**Table 4:** Results (positivity to *T. gondii*) of serological and molecular analysis, for each matrix and according to animal species.

Animal Species	No. Animals Analysed	ELISA Test			Real-Time PCR	
		Serum (No. Animals Positive)	Myocardium (No. Animals Positive)	Diaphragm (No. Animals Positive)	Myocardium (No. Animals Positive)	Diaphragm (No. Animals Positive)
Sheep	50	48	47	45	1	0
Goats	50	49	48	46	0	0
Cattle	45	6	8	7	0	0
Water buffaloes	48	4	3	1	0	0
Pigs	218	12	12	10	2	0

**Table 5:** Cohen’ ĸ values obtained from the comparison of the *T. gondii* antibody detection using different serological matrices (i.e., serum, meat juice from myocardium, meat juice from diaphragm) from different livestock species.

Animal Species	Serum vs. Myocardium (Cohen’s ĸ)	Serum vs. Diaphragm (Cohen’s ĸ)
Sheep	0.790	0.545
Goats	0.658	0.380
Cattle	0.831	0.910
Water Buffaloes	0.846	0.379
Pigs	1	0.904

*p* < 0.05 is statistically significant.

## Data Availability

Data are contained within the article or supplementary materials.

## Supplementary Materials

The following are available online at https://www.mdpi.com/article/10.3390/ani11071949/s1, Figure S1: Brochure designed to support the outreach activities. Figure S2: Poster designed to support the outreach activities.
