# Supplementary material for: Towards an Integrated Approach for Monitoring Toxoplasmosis in Southern Italy
Source: Animals (Basel). 2021 Jun 30;11(7):1949. doi: 10.3390/ani11071949 (PMC8300179; doi:10.3390/ani11071949)
Supplement: Supplementary file 1 [file animals-11-01949-s001.zip › FigureS2.pdf]

REGIONE CAMPANIA

C.Re.San.
CENTRO DI RIFERIMENTO REGIONALE
SANITÀ ANIMALE

TOXOPLASMOSI

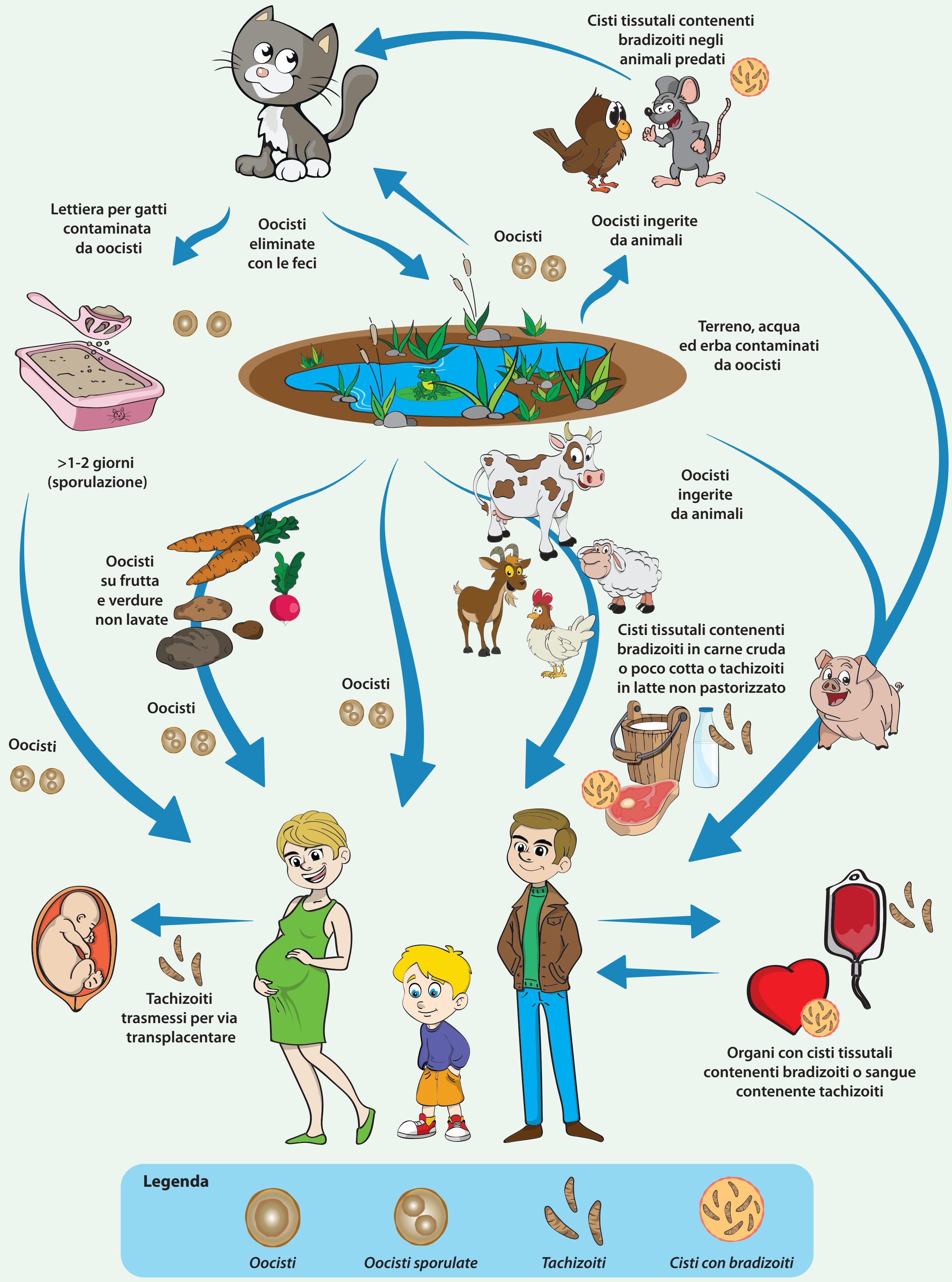

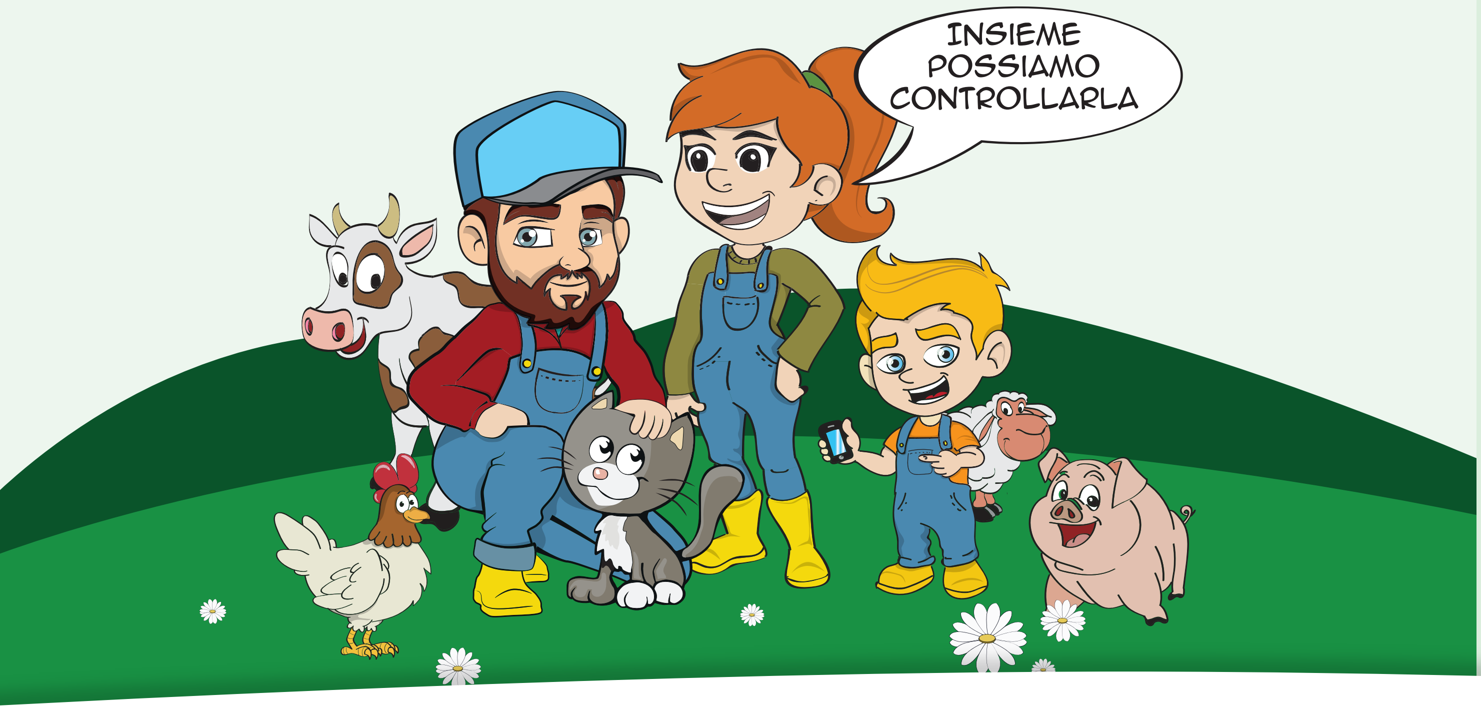

IL PARASSITA

Toxoplasma gondii è un parassita microscopico che colpisce diverse specie di animali e anche l'uomo. Questo microrganismo si presenta in tre forme biologiche diverse, tutte infettanti: le **oocisti** (nelle feci del gatto), i **tachizoiti** e le **cisti contenenti i bradizoiti** in organi e tessuti di molti animali e dell'uomo.

OSPITI DEFINITIVI E LOCALIZZAZIONE

In ambito domestico, il cosiddetto ospite definitivo di ***T. gondii*** è il gatto in cui il parassita si localizza nell'intestino e in vari organi e tessuti.

OSPITI INTERMEDI E LOCALIZZAZIONE

In ambito domestico molti animali sono sensibili all'infezione, i cosiddetti ospiti intermedi: ovini, caprini, bovini, bufali, suini, equini, cani, roditori e uccelli, nonché l'uomo. In tutti questi animali, e anche nell'uomo, il parassita si localizza in vari tessuti e organi: muscoli, fegato, polmoni, apparato riproduttore, cervello, etc. Anche il gatto è un ospite intermedio e, proprio perchè è sia ospite definitivo che ospite intermedio, viene anche definito ospite completo.

INFEZIONE

Ospite definitivo - Il gatto si infetta in vari modi: 1) predando topi e/o uccelli o ingerendo carni crude (o poco cotte) di animali infetti (ospiti intermedi); 2) per ingestione del parassita sotto forma di oocisti, eliminate con le feci da altri gatti.
Ospiti intermedi - Le vie di infezione degli animali ospiti intermedi sono diverse: 1) gli erbivori si infettano per ingestione del parassita sotto forma di oocisti eliminate dai gatti con le feci e presenti nel terreno, sugli alimenti (pascoli, mangimi, fieno), nell'acqua, etc; 2) i carnivori si infettano per ingestione di carni crude o poco cotte che contengono il parassita (sotto forma di cisti con bradizoiti); 3) in molti animali, soprattutto negli ovini e nei caprini, nei soggetti gravidi il parassita (sotto forma di tachizoiti) può attraversare la placenta ed infettare il feto. L'uomo si può infettare in tutti questi modi.

CICLO BIOLOGICO

Il complesso ciclo vitale (ciclo biologico) di ***T. gondii*** si può sintetizzare in due fasi:
Fase intestinale (solo nel gatto): il parassita si sviluppa nell'intestino e produce le cosiddette oocisti che vengono eliminate con le feci nell'ambiente esterno, dove maturano (sporulazione) e resistono per lungo tempo.
Fase extra-intestinale (tutti gli animali, uomo e gatto compresi): il parassita nell'organismo dell'ospite inizialmente attiva una moltiplicazione molto rapida producendo i cosiddetti tachizoiti che invadono organi, tessuti e sistema nervoso e possono attraversare anche la placenta di molti animali in gravidanza, anche della donna. In seguito, la reazione dell'ospite rallenta notevolmente la moltiplicazione del parassita che assume la forma di cisti (con all'interno i cosiddetti bradizoiti) all'interno degli organi, dei muscoli e del sistema nervoso degli animali e dell'uomo, dove rimangono molto a lungo, anche per tutta la vita dell'ospite.

SINTOMATOLOGIA

Il gatto di solito non manifesta sintomi. Anche nella maggior parte degli animali ospiti intermedi i sintomi dell'infezione spesso non sono evidenti. Gli ovini e i caprini sono invece gli animali maggiormente sensibili al parassita nei quali ***T. gondii*** causa principalmente aborto e mortalità neonatale. Nell'uomo la sintomatologia è variabile, da assenza di sintomi a forme gravi. Nelle donne in gravidanza la toxoplasmosi può avere conseguenze veramente molto gravi qualora l'infezione si realizza per la prima volta proprio durante la gravidanza: gli effetti possono essere aborto, malformazioni o gravi lesioni cerebrali del neonato.

RISCHIO PER L'UOMO

La principale via d'infezione per l'uomo è **la via alimentare** a seguito del consumo di carne cruda o poco cotta (soprattutto di ovini, caprini e suini), insaccati non ben stagionati, contenenti **le cisti con i bradizoiti**, del consumo di latte non pastorizzato (contenente **tachizoiti**) o di frutta e verdura crude o mal lavate contaminate dalle cosiddette **oocisti**. La manipolazione delle feci di gatto (o meglio della lettiera) espone molto all'assunzione di oocisti. Nell'uomo la trasmissione del parassita può verificarsi anche con la trasfusione di sangue intero o di globuli bianchi o a seguito di trapianto d'organo da donatore parassitato. Come già detto, nell'uomo esiste anche la possibilità di infezione per via transplacentare ovvero la madre trasmette l'infezione al feto in utero.

UNIVERSITÀ DEGLI STUDI DI NAPOLI FEDERICO II
Scuola di Agraria e Medicina Veterinaria
Dipartimento Medicina Veterinaria Produzioni Animali
Centro Regionale Monitoraggio Parassitosi CREMOPAR
Istituto Zooprofilattico Sperimentale del Mezzogiorno
